# Deleterious effect of Usutu virus on human neural cells

**DOI:** 10.1371/journal.pntd.0005913

**Published:** 2017-09-05

**Authors:** Sara Salinas, Orianne Constant, Caroline Desmetz, Jonathan Barthelemy, Jean-Marc Lemaitre, Ollivier Milhavet, Nicolas Nagot, Vincent Foulongne, Florence E. Perrin, Juan-Carlos Saiz, Sylvie Lecollinet, Philippe Van de Perre, Yannick Simonin

**Affiliations:** 1 Pathogenesis and Control of Chronic Infections, Université de Montpellier, INSERM, EFS, Montpellier, France; 2 BioCommunication en CardioMétabolique (BC2M), Université de Montpellier, Montpellier, France; 3 Institut de Médecine Régénératrice et Biothérapies, Université de Montpellier, CHU Montpellier, INSERM, U1183, Montpellier, France; 4 Plateforme CHU SAFE-IPS, Infrastructure Nationale INGESTEM, Montpellier, France; 5 Department of Bacteriology-Virology, CHU Montpellier, Montpellier, France; 6 MMDN, INSERM U1198, Université de Montpellier, Montpellier, France; 7 Department of Biotechnology, INIA, Madrid, Spain; 8 UPE, Anses Animal Health Laboratory, UMR1161 Virology, INRA, Anses, ENVA, Maisons-Alfort, France; University of Texas Medical Branch, UNITED STATES

## Abstract

In the last decade, the number of emerging Flaviviruses described worldwide has increased considerably. Among them Zika virus (ZIKV) and Usutu virus (USUV) are African mosquito-borne viruses that recently emerged. Recently, ZIKV has been intensely studied due to major outbreaks associated with neonatal death and birth defects, as well as neurological symptoms. USUV pathogenesis remains largely unexplored, despite significant human and veterinary associated disorders. Circulation of USUV in Africa was documented more than 50 years ago, and it emerged in Europe two decades ago, causing massive bird mortality. More recently, USUV has been described to be associated with neurological disorders in humans such as encephalitis and meningoencephalitis, highlighting USUV as a potential health threat. The aim of this study was to evaluate the ability of USUV to infect neuronal cells. Our results indicate that USUV efficiently infects neurons, astrocytes, microglia and IPSc-derived human neuronal stem cells. When compared to ZIKV, USUV led to a higher infection rate, viral production, as well as stronger cell death and anti-viral response. Our results highlight the need to better characterize the physiopathology related to USUV infection in order to anticipate the potential threat of USUV emergence.

## Author summary

Usutu virus (USUV) is an African mosquito-borne virus closely related to West Nile virus and belongs to the Japanese encephalitis virus serogroup in the *Flavivirus* genus. Recently several neurological disorders such as encephalitis, meningitis and meningoencephalitis were associated with USUV-infection in immunocompromised and immunocompetent patients. The goal of our work was to study the ability of USUV to infect neuronal cells and to characterize the effects of USUV infection in these cells. We have shown that USUV can infect efficiently several neuronal cells (mature neurons, astrocytes, microglia, IPSc-derived human neuronal stem cells (NSCs)). Interestingly, USUV replicates in human astrocytes more efficiently than another mosquito-borne flavivirus, Zika virus, reduces cell proliferation and induces strong anti-viral response. Moreover, USUV induces caspase-dependent apoptosis in NSCs. Our results suggest that USUV infection may lead to encephalitis and/or meningoencephalitis via neuronal toxicity and inflammatory response.

## Introduction

The recent Zika virus (ZIKV) outbreak has reminded us that the emergence of new viruses depends on multiple factors and is therefore extremely difficult to predict. Among potential emerging viruses, Usutu virus (USUV) has recently focused attention. USUV is an African mosquito-borne virus closely related to West Nile virus (WNV) that belongs to the Japanese encephalitis virus (JEV) serogroup in the *Flavivirus* genus (*Flaviviridae* family) [1]. USUV was discovered in 1959 from a mosquito of the *Culex neavei* species in South Africa and isolated by intracerebral inoculation of newborn mice [2]. The USUV genome is a positive, single-stranded RNA genome of 11,064–11,066 nucleotides with one open-reading frame encoding a 3434-amino-acid-residue polyprotein, which is subsequently cleaved into three structural (core, membrane, and envelope) and eight nonstructural (NS1, NS2A, NS2B, NS3, NS4A, 2K, NS4B, and NS5) proteins [3–5]. USUV natural life cycle is similar to WNV: it involves birds as reservoirs and ornithophilic mosquitoes as vectors like the common *Culex pipiens*. Notably, USUV-infected mosquitoes were recently detected in several European countries [6,7]. Mammals including horses or wild boars, were described as accidental or dead-end hosts [8–10].

Most of the sequences from USUV strains isolated in Europe can be differentiated from the originally isolated USUV strain SAAR 1776, whereas other sequences are closely related to the original isolated strain [11][12]. This suggests that USUV had been introduced in Europe several times from endemic areas in Africa, probably by migratory birds [12]. Interestingly, USUV has been detected in the wild (*i*.*e* common blackbirds (*Turdus merula*), waterfowls, raptors, greyland geese (*Anser anser*), mallard ducks (*Anas platyrhynchos*)) and domestic (sentinel chickens (*Gallus gallus domesticus)*, canary (*Serinus canaria domestica*)) birds in numerous European countries since 1996 [6,13–21]. Infected birds present severe neurological signs, often fatal, such as depression, incoordination and inability to fly [22]. These signs are associated with brainstem and cortical neuron necrosis [20,22]. In humans, USUV infection was described in Central African Republic and in Burkina Faso in 1981 and 2004 respectively, and associated with fever and skin rash [8]. Molecular and serologic evidences of USUV infection in Italian and German blood donor indicate that the virus is also silently circulating among asymptomatic humans in Europe and could thus be a concern for blood transfusions or organ transplants, as previously evidenced for the closely-related WNV [23–26]. Since 2009, some neurological disorders such as encephalitis, meningitis and meningoencephalitis were found associated with USUV-infection in immunocompromised and immunocompetent patients [27–30]. Importantly, a retrospective study published in 2017 showed that USUV was the cause of previously unexplained encephalitis in Italy suggesting that neurological cases associated to USUV may be more common than previously thought [31].

Although USUV is an emerging pathogen and dispersed quickly in Europe, very little is known about its pathogenesis, biologic features and host spectrum. It is nonetheless described that USUV infection upregulates the cellular autophagic pathway [32] and can induce type 1 IFN production [33,34]. Susceptibility of adult wild type (WT) mice to USUV is limited [35], whereas mice lacking the interferon type 1 receptor (*Ifnar1*^*-/-*^) are susceptible, as described for other flaviviruses such as ZIKV [36–39]. Experimental infections of 1-week-old suckling WT mice by intraperitoneal injection reproduced neurological signs such as depression, disorientation, paraplegia, paralysis and coma [40]. Spinal cord examination showed moderate neuronal death, especially in the ventral horns, and multifocal demyelination [40].

These observations highlight the urgent need for a clear understanding of the pathophysiological mechanisms involved in USUV infection, in particular in terms of neurovirulence and neuronal tropism. In this study, we used several cellular models to better understand USUV neuronal tropism and associated physiopathological effects. We demonstrated that USUV can efficiently infect murine mature neurons, astrocytes and microglia. We also compared the cellular effect of USUV with those of ZIKV, an emerging flavivirus that has been described to be link with neurological disorders including microcephaly and Guillain-Barré syndrome. ZIKV induces more rarely meningitis and encephalitis, and is thus consider to be less neuroinvasive than encephalitic flaviviruses in adults [41]. Here, we show that USUV infects human astrocytes more efficiently than ZIKV, reduces cell proliferation and induces stronger anti-viral response. Finally, we show that USUV strongly infect induced pluripotent stem cell (IPSc)-derived human neuronal stem cells (NSCs) and induces caspase-dependent apoptosis.

## Results

### USUV efficiently infects cells of the murine central nervous system

**Fig 1:**
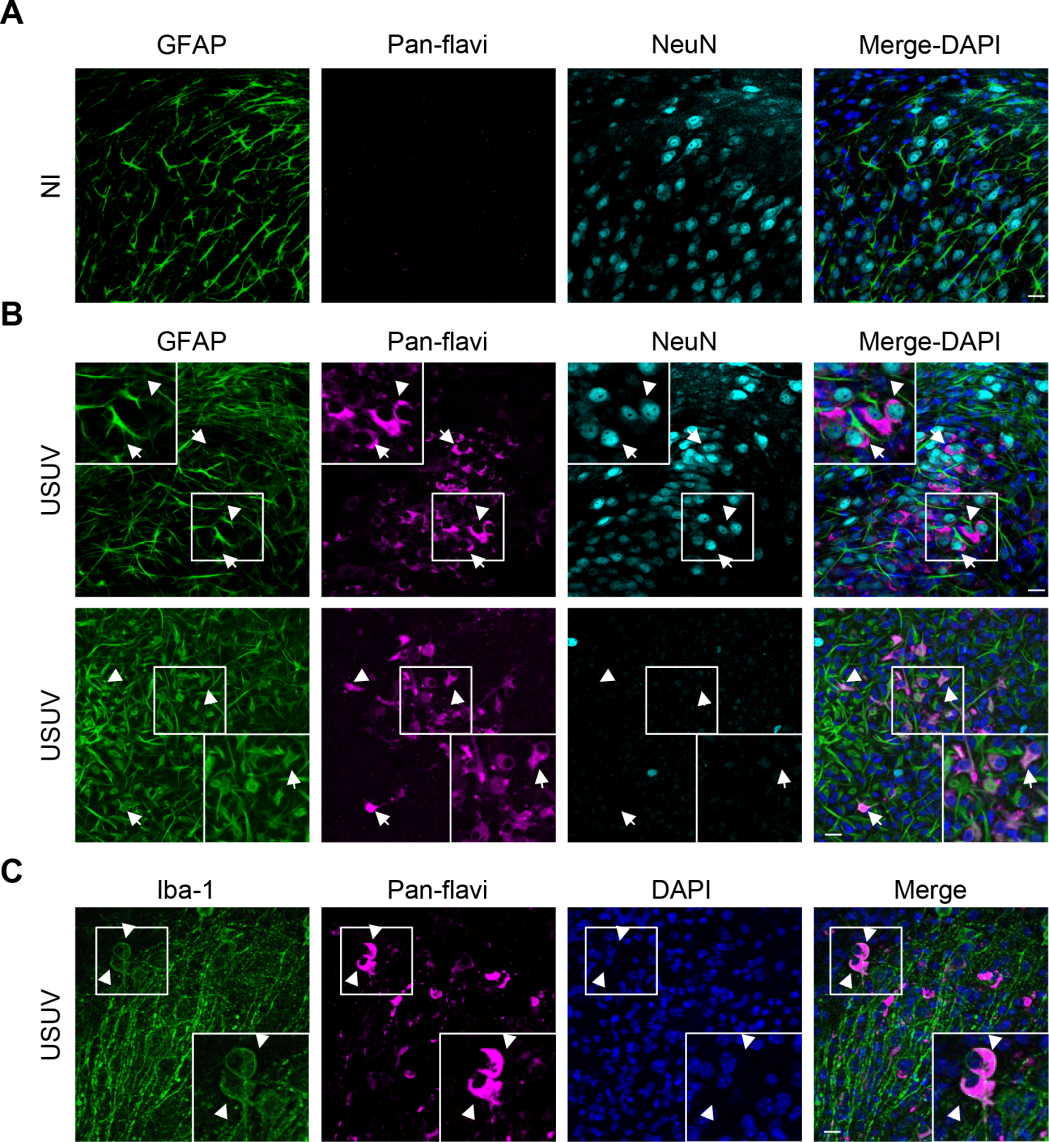
USUV infects efficiently organotypic murine brain slices. Hippocampi slices obtained from 6 day old pups were infected with USUV (3×10^5^ infectious particles per slice). Five dpi, slices were fixed and subjected for indirect immunofluorescence using various neural cellular markers such as GFAP (astrocytes), NeuN (neurons) and Iba1 (microglia). (A) Non-infected (NI) slices did not show labeling by the anti-pan-flavivirus antibody, in contrast to USUV-infected samples (in magenta) (B and C). (B and C) White arrows show infected cells also expressing either NeuN (in cyan), GFAP or Iba1 (in green), indicating that USUV can infect and replicate in multiple types of neural cells in the murine brain. Nuclei are labeled with DAPI (in blue). Zoomed in panels of white boxes show co-labelling. Scale bars 20 μm.

To better understand the involvement of USUV in neurological impairments, we first aimed to describe the cellular neurotropism of European strain of USUV (Vienna, 2001). Report of USUV-associated neuropathology in mice suggest that this animal model is pertinent to study USUV cellular interactions both *in vivo* and *ex vivo* [40,42]. To monitor viral replication in the murine central nervous system (CNS), we first used acute hippocampus slices prepared from dissected brains from 6–7 day-old wild type (WT) mice. Two days post-isolation, USUV was applied (3x10^5^ tissue culture infective dose 50% (TCID50) per slice) on top of the slices, which were further maintained in culture. 4 days post-infection (dpi), slices were fixed, astrocytes, microglial cells and neurons labeled by GFAP, Iba1 and NeuN staining respectively and USUV antigens were observed using a pan-flavivirus antibody (4G2) that recognizes the envelope protein of several flavivirus [43]. Fig 1A shows that in mock-treated slices, no pan-flavivirus labeling was observed, whereas USUV-infected samples showed strong pan-flavivirus staining, indicating an efficient USUV infection. Co-labeling with neuronal- (NeuN), astrocyte- (GFAP) and microglial- (Iba1) specific antibodies with the pan-flavivirus antibody showed a broad tropism of USUV for brain cells (Fig 1B and 1C).

**Fig 2:**
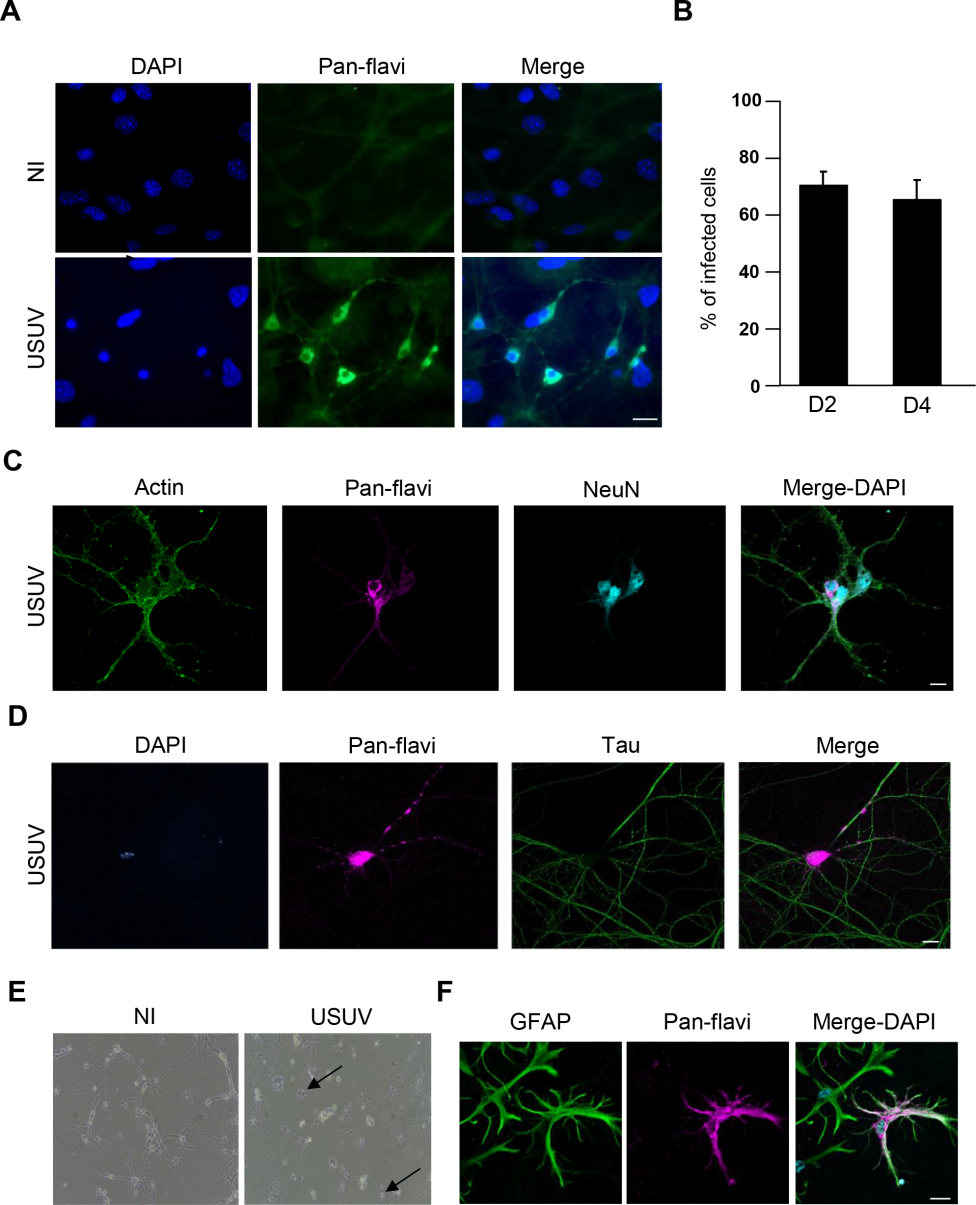
USUV infects multiple murine neural cells *ex vivo*. Primary murine hippocampal neurons were infected with USUV at a MOI of 2. (A) At 4 dpi, cells were fixed and labeled with a pan-flavivirus antibody. Strong labeling was observed in cells of neuronal morphology, both in soma and neurites. (B) Quantitative measurement showed that ~70% (+/- 4%) of cells of this quasi-pure culture were infected at day 2 and day 4 (n = 3 independent experiments). (C-D) USUV infects efficiently neurons *ex vivo*. Co-labeling from fixed USUV-infected cultures at 4 dpi demonstrates that USUV replicates in neuronal cell bodies (nuclei expressing NeuN) and axons (labeled with Tau). (E) USUV elicits neuronal toxicity. Bright light imaging of USUV-infected neurons at 8 dpi shows neurite damage. Arrows are showing dying cellular bodies. (F) Glia cells are infected by USUV. Cells expressing GFAP (astrocytes) were infected by USUV as shown by indirect IF studies at 4 dpi. Scale bars 20 μm.

To confirm these observations *ex vivo*, we infected quasi-pure primary hippocampal neuron cultures with USUV at a multiplicity of infection (MOI) of 2. Strong labeling was observed 4 dpi, with patches of viral proteins also found along neurites (Fig 2A). Quantification showed that around 70% of cells with neuronal morphology were efficiently supporting the replication of USUV at 2 and 4 dpi (Fig 2B). Supernatants from USUV-infected cells at 4 dpi were collected and applied to Vero cells to measure viral titer, quantified by tissue culture infective dose (TCID) 50. Efficient viral replication was observed with viral titer quantified around 1.6×10^6^ TCID50/ml while titer after inoculation was 1×10^4^ TCID50/ml. Similarly to what we observed in hippocampal slices, we detected viral antigens not only in mature neurons (labeled with NeuN, Fig 2C) and in cell bodies but also in axons (labeled with Tau, Fig 2D). Of note, neuronal damage was observed at late time post-infection, showing refringent cell bodies and neurite destruction in USUV-infected neurons at 8 dpi, suggestive of cellular toxicity (Fig 2E).

**Fig 3:**
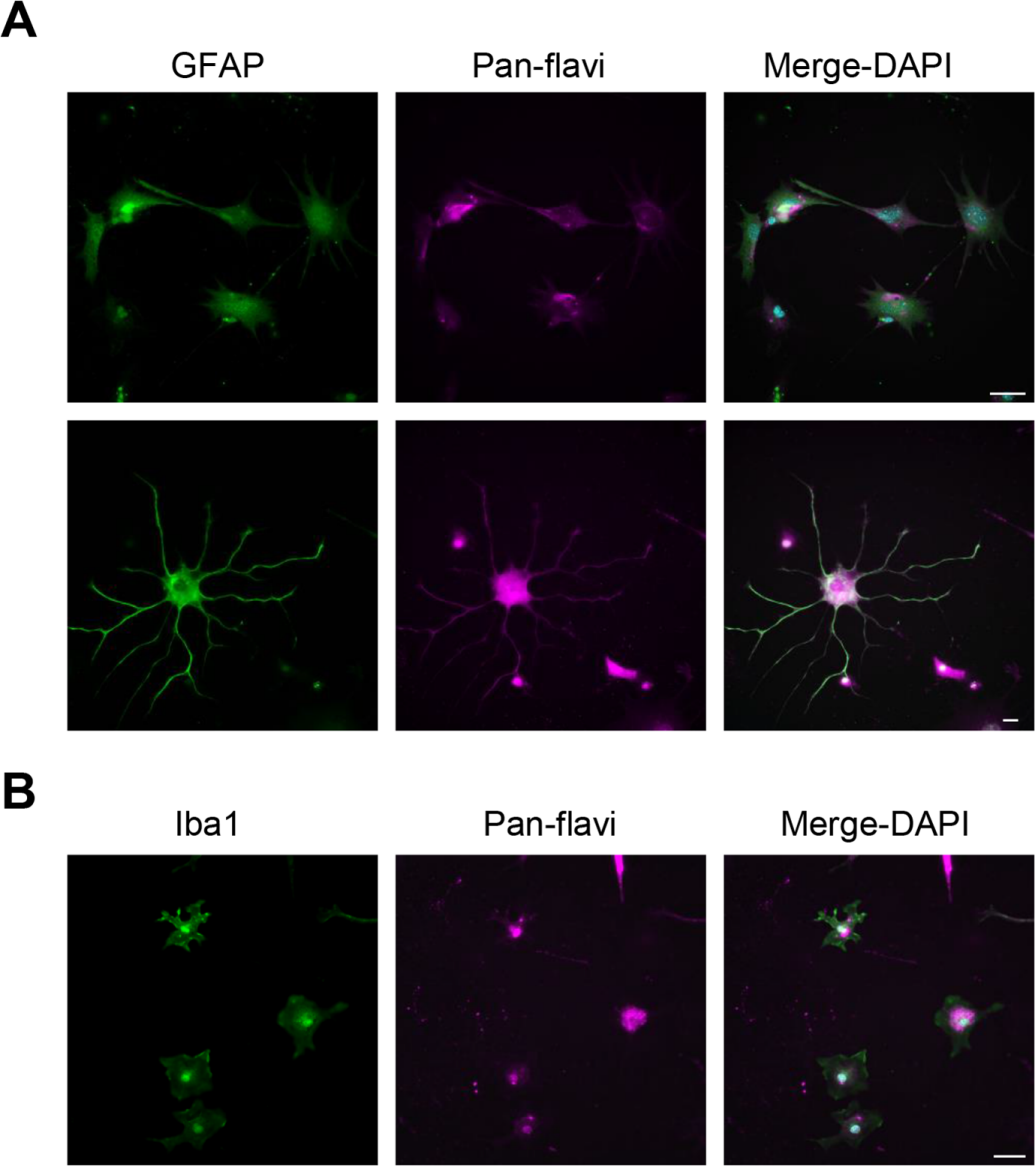
USUV replicates in murine spinal glial cells *ex vivo*. Spinal astrocytes and microglia are readily infected by USUV. Mixed cultures were infected with USUV at a MOI of 2 and fixed at 4 dpi. (A) USUV-infected cells labeled with the pan-flavivirus antibody also express GFAP, suggesting an efficient replication in spinal astrocytes. Rows show different pictures of the same experiment. Scale bar 10 μm. (B). USUV-infected cells labeled with the pan-flavivirus antibody express Iba1, suggesting an efficient replication in spinal microglial cells. Scale bar 20 μm.

Moreover, as the culture contains sparse glial cells, we observed that astrocytes were also infected as co-labeling pan-flavivirus and GFAP could be observed (Fig 2F). To study more precisely viral replication in glial cells, we took advantage of a culture of spinal glial cells that contains ~80% of astrocytes and ~20% of microglial cells. Four dpi, antigens were observed by immunofluorescence in GFAP- and Iba1-positive cells (Fig 3A and 3B) and viral titer of the supernatant was estimated as being around 9x10^6^ TCID50/ml while titer after inoculation was 2×10^4^ TCID50/ml.

Altogether, these data suggest a broad neurotropism for USUV in the murine CNS, possibly associated with neuronal toxicity, which could be consistent with the neurological disorders observed in animals and human.

### Primary human astrocytes are permissive to USUV infection

Extrapolating data observed in mice concerning cellular tropism or pathologies to human diseases can be misleading. Thus to better understand how USUV affects cells of the human brain, we first used primary human astrocytes. This cellular type is one of the first to be activated in brain infection and/or inflammation and are often targeted by flaviviruses including ZIKV [43] and WNV [44].

**Fig 4:**
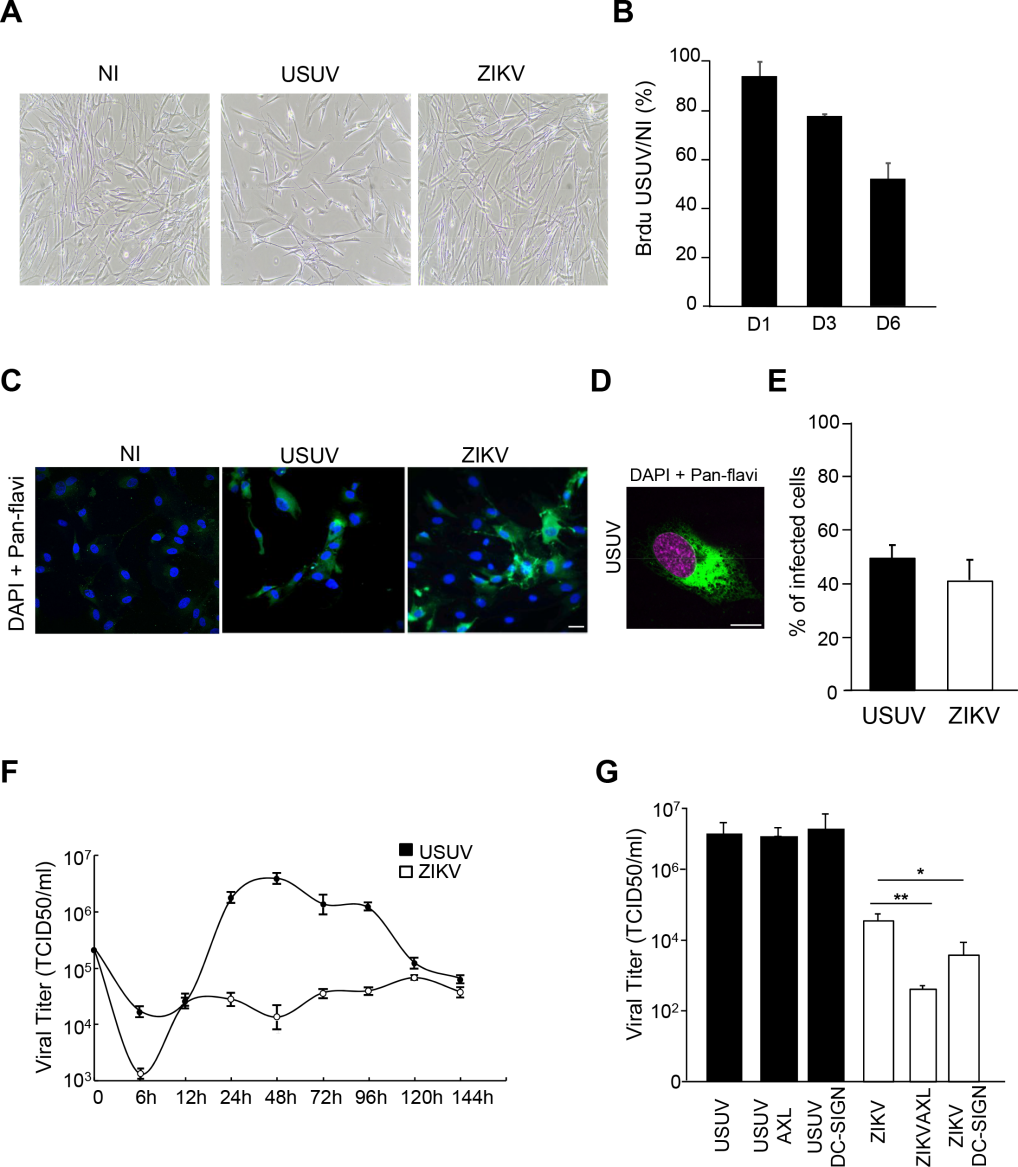
Comparative infections between USUV and ZIKV in primary human astrocytes. Primary human astrocytes were infected with USUV or ZIKV at a MOI of 2. (A) Bright light images of control and infected astrocytes at 4 dpi show sparser cells in USUV-infected condition. (B) Cellular proliferation is affected by USUV infection. Astrocytes pre-treated with BrdU were infected with USUV at MOI 2 and assayed for BrdU at 1, 3 and 6 dpi. (C) Mock, USUV- and ZIKV-infected cells were fixed at 4 dpi and labeled with the pan-flavivirus antibody (in green) by indirect immunofluorescence. Strong labeling was observed in cells. Nuclei are labeled with DAPI (in blue). (D) Typical flavivirus labeling (in green) is observed at high magnification. Nuclei are labeled with DAPI (false colored in magenta). (E) Quantification of USUV and ZIKV-infected cells. (F) Kinetics of viral production in USUV- or ZIKV-infected astrocytes show difference in terms of replication and persistence between the two viruses. Supernatants from infected cells (MOI of 2) were collected at various time points and subjected to TCID50 measurement on Vero cells. (G) AXL and DC-SIGN do not mediate USUV internalization in human astrocytes. Cells were pre-incubated with anti-Axl or Anti-DC-SIGN prior to infection with USUV or ZIKV at MOI of 2. 4 dpi, supernatants were collected and viral replication assayed. Blocking antibodies only decreased ZIKV replication. (*p<0.05, **p<0.01). Scale bar 10 μm.

Here, we aimed to compare astrocyte tropism/virulence of USUV and ZIKV, another Flavivirus recently described for its neurotropism and its ability to infect this cell type [45–49]. To this end, we infected astrocytes with USUV or ZIKV at a MOI of 2. We first monitored whether both viruses were inducing cytopathogenic effects (CPE) that are often characterized by cells rounding up in the process of dying by apoptosis, pyroptosis or necrosis. Four dpi, USUV-infected astrocytes appeared sparsely populated compared to non-infected and ZIKV-infected, and showed little if any CPE (Fig 4A). ZIKV did not appear to modulate cell proliferation or to trigger important CPE (Fig 4A). Importantly, upon USUV infection, at MOI of 0.1 and 2, we could not detect cell death, suggesting that apoptosis is not triggered in infected astrocytes (S1 Fig). Because bright light pictures of USUV-infected astrocytes seem to indicate a possible defect in cellular proliferation, we next investigated whether cell division was affected by USUV infection. The nucleotide analog BrdU was applied to astrocytes infected or not by USUV, and was quantified at 1, 3 and 6 dpi by ELISA. Compared to non-infected cells, USUV-infected astrocytes showed a decrease in proliferation from 3 dpi (Fig 4B). Moreover, we detected 48% infected cells by USUV and 41% by ZIKV using immunofluorescence pan-flavivirus staining, (Fig 4C and 4E), with a localization characteristic of the endoplasmic reticulum, a classical site for flavivirus replication (Fig 4D). Finally, supernatants from USUV or ZIKV-infected astrocytes (MOI 2) were collected at different time post-infection to measure growth kinetics (Fig 4F). For USUV, a plateau in replication was observed between 24 h and 96 h post-infection, followed by a decrease in viral titer. However, ZIKV viral titer was significantly lower than USUV and the plateau lasted longer (Fig 4F).

Finally, the proteins AXL and DC-SIGN have been reported to act as cellular receptors for some flaviviruses, including ZIKV [50–52]. To monitor whether USUV is using these proteins to infect astrocytes, we performed a competition experiment by pre-incubating cells with either anti-AXL or anti-DC-SIGN antibodies prior to infection with USUV or ZIKV. By measuring viral titer 4 dpi, we confirmed that blocking AXL decreased ZIKV replication (Fig 4G), while blocking DC-SIGN impaired to a less extent ZIKV replication in astrocytes (Fig 4G). In contrast to ZIKV, USUV replication was not modulated by blocking either molecules, suggesting that USUV does not act through these specific cellular receptors to infect human astrocytes (Fig 4G).

The data demonstrate that USUV is not only more efficiently targeting human astrocytes than ZIKV but also may trigger deleterious effects by acting, at least partially, on cellular proliferation.

### USUV infection of human astrocytes elicits a strong anti-viral response

Because both USUV and ZIKV efficiently infected primary human astrocytes, we next aimed at analyzing the modulation in the expression of genes involved in anti-viral responses. We used a PCR array consisting in 84 genes that are modulated in the interferon (IFN) response or the cellular pattern recognition receptors (PRR) among other mechanisms [43] (S1 Table).

**Fig 5:**
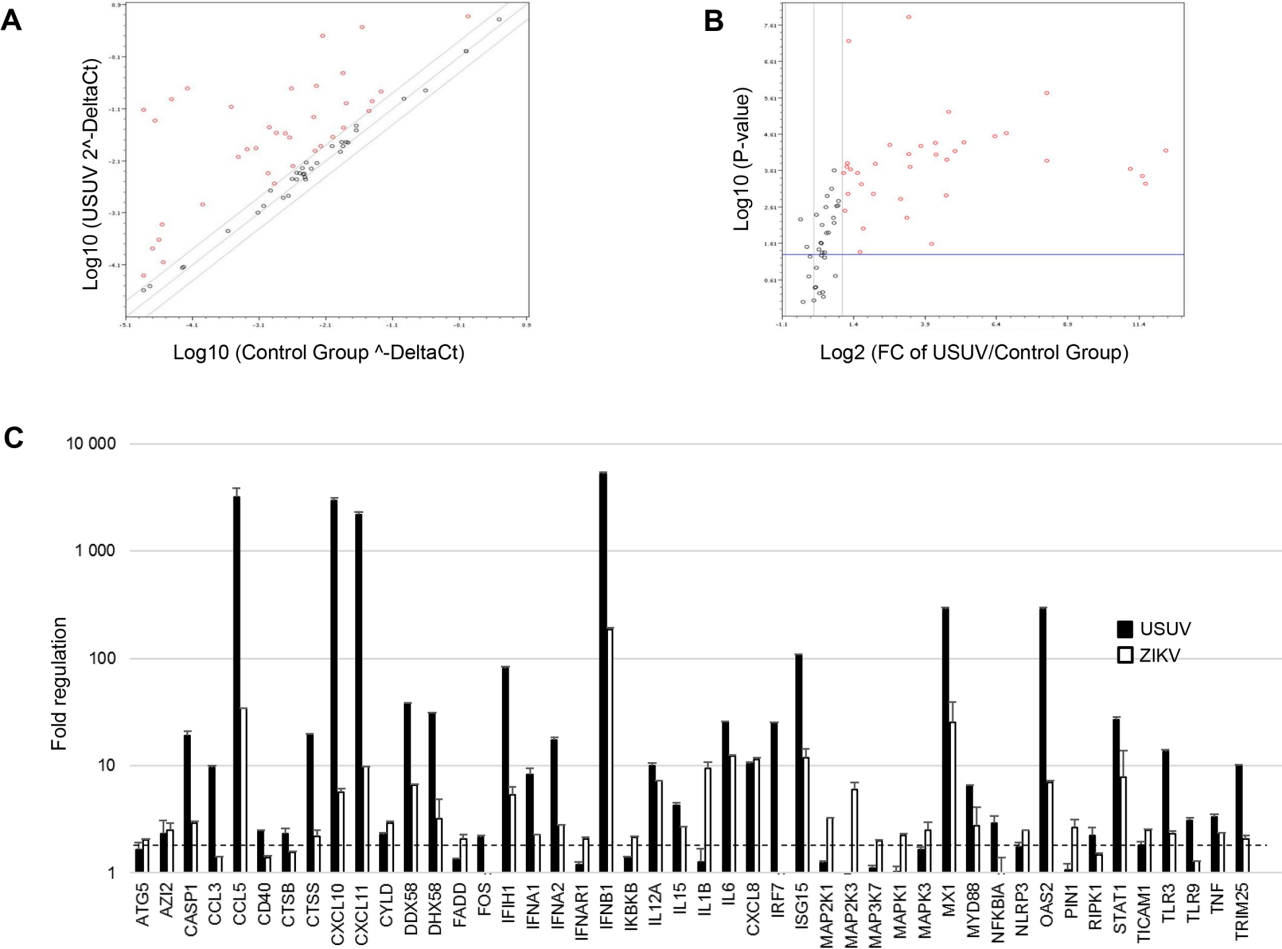
Anti-viral responses in USUV- and ZIKV-infected human astrocytes. mRNA from supernatants of primary human astrocytes infected with USUV or ZIKV at MOI = 1 for 3 days were subjected to qRT-PCR analyses. (A) Scatter plot. Up regulated genes appears in the top-left cadran (boundary 2). (B) Volcano plots, (boundary 2, scatter 0.05: *p*-values based on student’s t-test of three replicates). Statistically significant changes in fold regulation appear in the top right part of the graph (genes upregulated). The experiment was performed in triplicates and each point represents the mean. (C) Fold regulation of statistically significant genes modulated upon USUV and ZIKV infection are shown. Results are expressed as means ± SD and analyzed using an unpaired *t*-test *p <0.05.

To compare the anti-viral response elicited by flavivirus infection in astrocytes, mRNAs were collected from USUV- and ZIKV -infected astrocytes 4 dpi and subjected to retrotranscription. cDNA relative abundance was then analyzed by qPCR. Under these conditions, we found that 33 genes were significantly upregulated (more than 2 fold) by USUV in astrocytes (Fig 5A–5C). Several cytokines and chemokines genes were found upregulated upon both USUV and ZIKV infections such as *IFN-Β*, *TNF*, *IL12A*, *IL15*, *IL6*, *CCL5*, *CXCL10*, *CXCL8* OR *CXCL11*. Moreover, gene expression of PRR genes such as *Ddx58* (RIG-I), *Dhx58* (LGP2) or *Tlr3* were also upregulated by both viruses, whereas *Tlr9* was only modulated by USUV (Fig 5C). Importantly, in all cases, the upregulation of these antiviral genes was stronger following USUV than ZIKV infection, up to 100 times for the chemokines *CCL5*, *CXCL10* and for the *IFN-Β* (Fig 5C). Other genes, such as the chemokine *CCL3*, *CD40*, *CTSB* and the transcription factors *FOS* and *IRF7* were specifically upregulated in USUV infected astrocytes. Interestingly, other differences in the cellular responses triggered by USUV *vs* ZIKV were observed, in particular regarding the MAPK pathway (*MAP2K1*, *MAP2K3*, *MAP3K7*, *MAPK1*, *MAPK3*), the peptidyl-prolyl isomerases *PIN1* and members of the inflammasome pathway such as *Nlrp3* and *Il1β* that were preferentially modulated by ZIKV (Fig 5C).

These data highlight the strong induction of an antiviral response by USUV and suggest substantial differences in the cellular response process against flaviviruses in astrocytes.

### Human IPSc-derived NSCs are undergoing apoptosis under USUV infection

**Fig 6:**
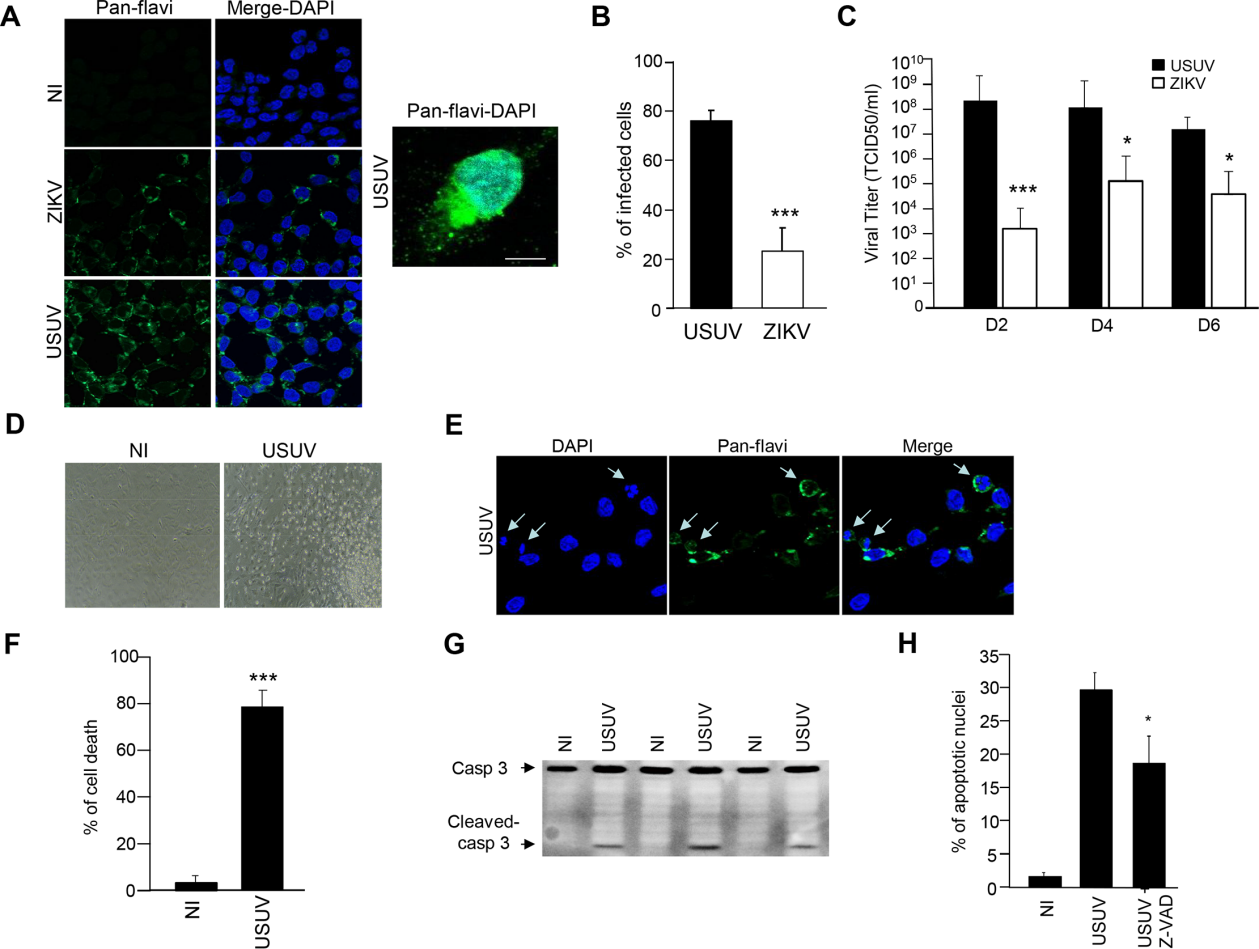
Effect of USUV infection on NSC survival. (A) IPSc-derived NSCs were infected with USUV and ZIKV at a MOI of 2 and fixed at 2 dpi. Cells were labeled with the pan-flavivirus antibody (in green) by indirect immunofluorescence. Nuclei are labeled with DAPI (blue). Scale bar 10 μm. (B) Quantification of the percentage of USUV- and ZIKV-infected cells. (C) Supernatants from USUV- and ZIKV infected NSCs (MOI 2) were collected at 2, 4 and 6 dpi and subjected to TCID50 measurement on Vero cells. (D) Bright light images of control and USUV-infected NSCs at 4 dpi show rounded up cells in USUV-treated condition. (E) USUV-infected NSCS at 4 dpi and labeled with the anti-flavivirus antibody and DAPI show condensed nuclei. (F) Trypan blue assay (supernatant + adherent cells) showed that cell viability is affected in USUV-infected NSCs at 4 dpi. (G) Immunobloting analyses of cell extracts from mock or USUV-infected cells at 4 dpi show the generation of cleaved caspase-3 fragment, indicative of apoptosis. (H) NSCs were infected with USUV at a MOI of 2 with or without Z-VAD. 4 dpi, cells were labeled with the anti-flavivirus antibody and DAPI and condensed nuclei quantified. (*p<0.05, ***p<0.001).

The recent ZIKV epidemic highlighted that developing brains can be highly sensitive to flavivirus infection [45–48,53,54]. Moreover, in the adult brain, specific niches such as the hippocampus are involved in adult neurogenesis and are potential targets for viral infections [53]. To study the potential tropism for USUV to human NSCs, we used IPSc-derived NSCs obtained according to standard methods [43]. NSCs were infected with USUV and ZIKV at a MOI of 2 and efficient USUV and ZIKV infectivity were detected 2 dpi using the pan-flavivirus antibody (Fig 6A). Moreover, we detected 77% infected cells by USUV and only 21% by ZIKV using immunofluorescence pan-flavivirus staining (Fig 6B). Production of infectious particles was observed as attested by viral titers of approximately 10^8^ TCID50/ml obtained from supernatants of USUV-infected NSCs, which was significantly higher than ZIKV at 2, 4 and 6 dpi (Fig 6C). Interestingly, and in contrast to astrocytes, USUV-infected NSCs at 4 dpi showed round-up morphology and condensed nuclei, suggestive of an apoptotic cell death (Fig 6D and 6E). Quantification of cell viability by trypan blue showed that ~80% of USUV-infected NSCs were indeed undergoing cellular death at 4 dpi (Fig 6F). Similarly, activated-caspase 3 was observed by immunoblotting, confirming that USUV infection triggered apoptotic pathways in IPSc-derived NSCs (Fig 6G). USUV-associated cellular death could be strongly decreased by pre-treating NSCs with the anti-apoptotic agent Z-VAD (pan-caspase inhibitor) prior to USUV infection. Indeed, NSCs treated with Z-VAD for 4 days concomitantly to USUV showed fewer apoptotic nuclei than USUV-infected cells without Z-VAD treatment (Fig 6H).

Altogether, these observations suggest that NSCs are strongly permissive to USUV infection and undergo cellular death by caspase 3-dependant apoptosis.

## Discussion

Despite its immune-privileged status, the CNS can respond efficiently to viral challenges. Following CNS infection, homeostasis can be altered as inflammation can arise in distinct anatomical regions causing inflammatory diseases such as myelitis, meningitis, encephalitis and meningoencephalitis that can severely affect human health and be associated with long-term sequelae. Symptoms and severity of the disorders caused by neurotropic viruses depend on several factors, including cell tropism, viral cytopathogenicity and the host immune response.

Numerous flaviviruses have been described to access to the CNS and cause neuronal impairment [55], in particular Dengue virus (DENV), Japanese encephalitis virus (JEV), Tick‑borne encephalitis virus (TBEV), WNV and ZIKV, [56,57]. JEV, TBE and WNV are particularly neurovirulent and can cause encephalitis in humans. In contrast, DENV is more viscerotropic [58] and ZIKV appears to be less neuroinvasive in adults in comparison to the typical encephalitic flaviviruses [41]. Neurotropic viruses are continually emerging and are particularly problematic because they are often less adapted to their new host, and they can spread rapidly in the population and induce severe disorders, as experienced during the recent ZIKV epidemics. In this study, we report for the first time that USUV is able to infect efficiently a wide range of neuronal cells *ex vivo* (mature neurons, astrocytes, microglia and IPSc-derived human NSCs) associated with deleterious effects. The neurotropic ability of USUV was first suspected in birds since strong avian mortality has been described in Austria in 2001 [20], whereas the ability of the virus to infect humans is known since 1981 with a case in Central African Republic [59]. In 2009, a severe meningoencephalitis case related to USUV infection was reported in an immunocompromised Italian patient, demonstrating the zoonotic potential of USUV [27]. An additional case was also detected after orthotropic liver transplantation [28]. Later, a study in Croatia revealed also the ability of USUV to infect immunocompetent patients as three additional USUV neuroinvasive infections in humans (meningitis and meningoencephalitis) were described [29,30]. In 2016, a retrospective study detecting USUV RNA and USUV antibodies in cerebrospinal fluid (CSF) and serum samples revealed the presence of anti-USUV antibodies in more than 6% of samples analyzed in Italy [60]. Moreover, the authors detected USUV RNA in eight cerebrospinal fluids (CSFs). Among them, four patients were identified with neurological symptoms (encephalitis and meningoencephalitis). This last study suggest that USUV infection in humans may be more common than previously thought, at least in specific areas, and highlights the need to better understand the pathophysiology of this virus.

Neurons are a direct or indirect target for neurotropic flaviviruses. Mechanisms of neuronal injury after viral infection could be explained by several non-exclusive mechanisms such as direct cell death, destruction of infected cells in the CNS by cytotoxic T lymphocytes or neuronal cell death or dysfunction by bystander-infected cells. Notably, as USUV seems to infect different types of CNS cells (neurons, microglia and astrocytes), we could expect a combination of mechanisms that ultimately could lead to neuronal dysfunction. WNV, a closely related flavivirus that is also associated with encephalitis and other neurological disorders, has also been described to target neurons in the CNS, leading to their alteration or death (by caspase-3 driven apoptosis) [61]. In addition to direct damage, WNV infection may also trigger apoptosis in neurons as a result of bystander effects caused by cytotoxic factors released by (dying) neuronal and non-neuronal cells as it can also infect astrocytes [44]. Neuroinvasion does not seem to be a major feature of ZIKV virus infection as only few cases of encephalitis and meningoencephalitis have been reported for ZIKV. Studies indicate that it can poorly infect mature neurons, suggesting that its effects in the adult CNS could be mostly due to infection of glial cells such as astrocytes [51]. Following flavivirus infection activated glial cells release TNF, IL1β, IL6, and RANTES, all of which promote bystander damage to neurons. However, while the extent to which glial infection contributes to JEV-induced neurological disease has been well studied, its relevance in WNV-induced disease has received less attention [62]. Our data show that USUV can efficiently infect mature neurons that undergo cell death at late stage of infection. Resident cells of the CNS have developed innate immune antiviral strategies to defend against neurotropic viruses. While neurons are a target of neurotropic flaviviruses, other cell types (*e*.*g*., astrocytes, microglia, oligodendrocytes) might also be infected and contribute to the resolution of infection by generating immune responses against viral infections. Astrocytes are one of the most abundant cell types in the brain and spinal cord and mediate diverse supportive functions and immune regulation. Activated astrocytes produce a wide variety of cytokines and chemokines, including IFNs [63]. In our study, we found that primary human astrocytes respond to USUV infection by strongly upregulating chemokines and cytokines. The inflammatory profile is partially different following ZIKV infection, which is mainly characterized by less inflammatory response and by the specific induction of several MAPK genes (*MAP2K1*, *MAP2K3*, *MAP3K7*, *MAPK1*, *MAPK3*). Notably, we observed upregulated mRNA expression levels of *IFNα/β*, *IL6*, *TNF* and several chemokines as *CXCL10* or *CXCL11* after USUV infection. Generally, innate immunity is mandatory for clearance of viral infections [64], and when clearance is inefficient, an exaggerated cytokine release could be detrimental and associated with adverse effects such as cancers or CNS disorders [65]. Therefore, the highest risk during neurotropic viral infection is the spread of the virus to the CNS, causing induction of inflammatory responses and the destruction of neuronal cells. For example IL-6 and TNF production by astrocytes can lead to increased permeability of the BBB [66], moreover CXCL10 has been reported to induce neuron apoptosis or direct damage in neuronal cells [67,68] and has been described to be activated in astrocytes after WNV infection [69]. Notably, we found that USUV induces astrocyte proliferation arrest. This phenotype could be related to the strong immune response observed after USUV infection. Indeed, some cytokines such as IFN-β and CXCL10 are known to cause cell death directly or to inhibit cell proliferation [70,71]. As astrocytes are essential for providing trophic support to neurons and maintain synaptic functions, loss of astrocytes can induce significant neuronal dysfunction and damage. In fact, when activated in an uncontrolled manner, astrocytes can release various substances, such as reactive oxygen species and inflammatory cytokines, triggering the cascade of events leading to neuronal degeneration. The most notable inflammatory response following USUV infection is the very strong upregulation of IFNβ (>7000 times). Interestingly, this strong IFN-β induction did not prevent USUV replication. These findings further support the hypothesis that USUV do not possess mechanisms that interfere with IFN induction as previously suggested in other studies on dendritic and epithelial cell [33,34]. The type I IFN response can limit viral dissemination by different mechanisms: restricting the spread of progeny viruses to neighboring cells or/and reducing overall viral replication. In this context, USUV infection inhibits IFN antiviral activity through a mechanism that remains to be determined and that could allow USUV to overcome this response to establish a productive infection. The absence of efficient protective effect of type 1 IFN has been demonstrated for other neurotropic flavivirus. For example in the case of DENV, type I IFN response limits only initial viral replication but has no apparent effect on controlling the virus from the CNS and disease development [72]. Similarly, functional type I IFN response was not protective against lethal encephalitis during WNV infections [73]. Moreover, there are several examples demonstrating that flaviviruses produce effective immune modulatory proteins and use multiple immune evasion mechanisms that limit host immune responses and favor viral replication [74,75]. Indeed, viruses possess specific mechanisms to subvert IFN antiviral effects through proteins that mimic and interfere with host proteins by delaying the interaction of pathogen-associated molecular patterns (PAMPs) with the cellular PRRs, suppressing the IFN-signaling or impairing functions of antiviral ISG. Thus, it could be hypothesized that the association of USUV with human diseases, such as encephalitis or meningitis, could be linked to the inability of the virus to suppress type I IFN production combined with the ability of the virus to overcome response in infected cells.

Finally, several receptors have been reported to facilitate flavivirus entry, including DC-SIGN, a type 2 transmembrane C-type lectin and AXL that belongs to the Tyro3 AXL Mer (TAM) family, a group of tyrosine kinase receptors [76]. AXL is known to be present in brain cells, including radial glial cells, astrocytes, and microglial cells [77]. We observed that the blocking antibody anti-AXL, and in a lesser extend anti-DC-SIGN, inhibits ZIKV replication in human astrocytes as previously described for AXL in human glia cells [50–52]. However, neither blocking AXL nor DC-SIGN has effect on USUV replication, suggesting that this flavivirus uses other entry receptor(s) in astrocytes that remain to be identified.

Recent observations showed that some flavivirus as ZIKV infection can also impair neurodevelopment while other neurovirulent Flaviviruses such as JEV and WNV are rarely linked to congenital malformations. Indeed, numerous studies demonstrated that ZIKV can infect human cortical NSCs, attenuates their proliferation and induce apoptosis, both in monolayer culture and in cerebral organoids or neurospheres [45–48,53,54]. In this study, we show that USUV can also infect neuronal progenitors with high efficiency and induces massive caspase-dependent apoptosis. A previous study showed that deleterious consequences of ZIKV infection in human NSCs are not a general feature of the flavivirus family, as this effect was not observed with DENV [47]. Our results show that USUV has also the ability to infect human NSCs *ex vivo*. It remains to be determined if USUV can access the fetal brain, but in regards of our results more investigations are necessary to investigate whether USUV infection can cross placental and blood brain barrier and have an impact during different stages of fetal development.

In conclusion, we showed that USUV is capable of infecting mature neurons, microglia, human neuronal precursors and primary human astrocytes. Whereas USUV infection kills neurons and NSCs, astrocyte infection causes cell proliferation arrest and induction of cytokines and chemokines. Our findings suggest that USUV infection may lead to encephalitis and/or meningoencephalitis via neuronal destruction and inflammatory response. There results, and the recent observations that USUV circulation in human may be more common than previously thought, highlight the need to include USUV in the differential diagnosis of encephalitis/meningoencephalitis cases of unknown etiology in areas where the virus is known to circulate. A better understanding of the epidemiological and biological characteristics of USUV infection is necessary to provide tools for anticipating the potential threat of USUV emergence.

## Materials and methods

### Antibodies

Antibodies used in this study are: anti-pan-flavivirus (MAB10216, clone D1-4G2) anti NeuN (Abcam), anti Iba-1 (Abcam), anti Tau (Abcam), anti-GFAP (Abcam), anti-caspase 3 and anti-activated caspase 3 (Cell Signalling Technology) and secondary antibodies coupled to Alexa dyes (488, 555 or 647, Thermofischer Scientific).

### Treatments

One hour before infection, cells were treated with AXL-blocking antibody or goat IgG control at 10μg/mL and with DC-SIGN at 5μg/mL (R&Dsystems). For apoptotic test cell cultures were incubated with or without zVAD-fmk (100 μM) (Abcam) for 90 min before infection and maintained during all the infection process.

### ZIKV and USUV strains, production and cellular infection

PF-13 ZIKV was produced and provided by the National Reference Center for arboviruses (NRC) and has no more than 5 passages on Vero cells (ATCC). USUV Vienna Austrian strain of USUV (Vienna2001-blackbird, USUV 939/01, GenBank acc: AY453411.1), was provided from Department of Biotechnology, INIA Madrid and was propagated three times in Vero cells. Viral stocks were prepared by infecting sub confluent Vero cells at the multiplicity of infection (MOI) of 0.01 in D-MEM medium (Thermoscientific) supplemented by 2% heat-inactivated fetal bovine serum (Sigma). Cell supernatant was collected 6 days later and viral stock harvested after centrifugation at 300 *g* to remove cellular debris. Viral titers were determined by the 50% tissue culture infective dose (TCID50), which was calculated using the Spearman-Kärber method [78] and were expressed as TCID50 per mL.

Cells at 60–70% confluence were rinsed once with phosphate-buffered saline (PBS), and ZIKV and USUV diluted to the required MOI was added to the cells in a low medium volume. Cells were incubated for 2 h at 37°C with permanent gentle agitation and then culture medium was added to each well, and cells were incubated at 37°C and 5% CO_2_. As control, cells were incubated with the culture supernatant from Vero cells (mock condition).

### Organotypic murine brain slices

All pups were anesthetized prior to brain extraction. Briefly, hippocampi from 6- to 7-day-old mice (Janvier, France) were dissected under aseptic conditions and transverse sections were obtained using a tissue chopper. Slices were placed on a 30-mm porous membrane (Millicell-CM) and kept in 100-mm diameter dish. Petri dishes were filled with 5 ml of culture medium composed of 25% heat inactivated horse serum, 25% Hank’s Balanced Salt Solution (HBSS), 50% minimum essential medium (MEM), 25 U/ml penicillin, 25-μg/ml streptomycin (Invitrogen). Cultures were maintained in a humidified incubator at 36°C and 5% CO_2_. Two days later, media were changed and the temperature set to 33°C.

### NSC generation and maintenance

NSCs were obtained from the SAFE-IPSc platform at IRMB (http://www.chu-montpellier.fr/fr/chercheurs/plateformes/les-plateformes-recherche/safe-ips/). Briefly, iPSCs were individualized with Gentle Cell Dissociation Reagent (Stemcell, 07174). They were rinsed out with Dulbecco’s modified Eagle’s medium/Ham’s F12 (DMEM/F-12, Gibco, 31330038) and centrifuged at 300 *g* for 5 min. Dissociated cells were plated on matrigel at a density of 20,000–40,000 cells/cm_2_ and cultured in neural induction medium (Stemcell, 05835) supplemented with 10 μM ROCK-inhibitor (Y-27632). Cells were allowed to reach 80–90% confluence over 6 days. Medium was changed daily with neural induction medium without Y-27632. IPSc-derived NSCs were passaged by incubation with trypsin at 0.005% to allow dissociation, and then seeded on poly-D-ornithine/laminin coated plates at 20,000 cells/cm_2_ in 50% DMEM/F-12 and 50% Neurobasal medium (Thermoscientific) supplemented with 1X N2 (Thermoscientific), 1X B27 (Thermoscientific), glutamax (Thermoscientific) and β-FGF plus EGF (Peprotech, 20 ng/mL each). The medium was changed every two days. Cells were used between passage 5 and 8.

### Astrocytes culture

Astrocytes were purchased from ScienCell^TM^ and cultured according to the manufacturer’s instruction. Cells were cultured on poly-D-lysine coated plates and were used between passage 4 and 8. Cell proliferation was assessed seeding 5000 cells in 96 well plates. At days 1,3,6 a bromodoxyuridine ELISA assay was performed (Calbiochem BrDU cell proliferation assay) following manufacturer instructions. Absorbance at 450 nm was measured using a spectrophotometer (TECAN).

### Hippocampal neurons culture

Mouse hippocampal neurons were obtained from OF1 embryonic day 18 (E18) embryos using standard procedures (Harlan). Briefly, hippocampi were isolated, dissociated with 0.025% trypsin and plated in Neurobasal medium (ThermoFischer) containing B27 (ThermoFischer), L-glutamine (Sigma), Glutamax (ThermoFischer), 10% fetal bovine serum (FBS, Sigma) and antibiotics. Hippocampal neurons were then incubated at 37°C and 5% CO2 under a humidified environment. At day *in vitro* (DIV) 4, 2/3r of the medium was replaced with medium without L-glutamine and FBS. Neurons were used at DIV8-10.

### Glial cell culture

Primary cultures of glial cells were established from the spinal cord of 16-day-old C57/Bl6 mice (Harlan). Animals were sacrificed in aseptic conditions. Spinal cords were dissected, freed from meninges and collected in cold HBSS supplemented with calcium and magnesium (Gibco), glucose (Sigma, 6 g/L) and 1% antibiotic solution (penicillin/streptomycin, Gibco). Tissues were chopped and rinsed (3 times) in HBSS (without calcium and magnesium, 1% antibiotic solution), supernatant was removed. Tissues were re-suspended in 1.5 ml of DMEM/F12 medium (Invitrogen) and 1% penicillin/streptomycin and treated with 2 ml 0.25% trypsin-EDTA (Gibco) for 20 minutes at 37°C. Trypsin was inactivated by adding 10% FBS. DNAse1 (10mg/ml, Rotkreuz. Switzerland) was then added. Cells were mechanically dissociated and re-suspended in 10ml of culture medium consisting of DMEM/F12, glucose 6 g/L, glutamax 100X (Gibco), 10% FBS (Gibco) and 1% penicillin/streptomycin. Centrifugation (5min, 500g, room temperature) was done and supernatant removed. Cells were re-suspended in the same culture medium and plated at a final concentration of 50 000 cells/well on glass coverslips treated with 25 μg/ml of low-molecular weight poly-D-lysine (Sigma-Aldrich, St Louis, MO) in 24-well dishes (Nunc, Roskilde, Danemark).

### Immunofluorescence assays

NSCs were plated on poly-D-ornithine/laminin coverslips and astrocytes plated on poly-D-lysin coverslips. For indirect immunofluorescence, cells were fixed with 4% PFA and permeabilized with 0.1% Triton X-100/PBS for 5 min at room temperature (RT), followed by a blocking step with 2% bovine serum albumin (BSA) and 10% horse serum for 30 min to 1 h at RT. Primary and secondary antibodies were diluted in blocking solution and incubated sequentially for 1h at RT. Samples were then mounted with fluorescent mounting medium (Prolongold, Thermofischer) with DAPI (Sigma) and imaged by confocal microscopy using the Zeiss SP85 confocal microscope, with 40× or 63× 1.4 NA Plan Apochromat oil-immersion objectives.

### Immunoblotting

Cells were lysed by boiling in SDS sample buffer, sonicated, and complemented with dithiothreitol (DTT). Protein concentrations were measured by a bicinchoninic acid (BCA) protein assay kit (Pierce, MA, USA). Equal amounts of protein from total cell lysates (10 μg) were loaded on SDS-PAGE gels and transferred onto nitrocellulose membranes. The membranes were blocked and incubated overnight at 4°C with primary antibodies and then incubated with horseradish peroxidase (HRP)-conjugated secondary antibodies (Amersham) for 1 h, bands were visualized by ChemiDoc XRS plus (Biorad Laboratories Hercules, CA).

### RT-qPCR

Astrocytes infected with USUV and ZIKV or mock-treated cells were harvested in RLT buffer (Qiagen). Total RNA was extracted using RNeasy mini-kit (Qiagen). Complementary DNA was synthesized using Omniscript reverse transcriptase (Life Technologies). RT2 Profiler PCR arrays for Human Antiviral Response (96 well format, Qiagen) were used for real-time quantitative PCR analysis, with the use of the LC480 real time PCR instrument (Roche) and the Light Cycler 480 SYBR Green I master Mix (Roche). Volumes of mix, cDNA, RNAse-free water, and cycling conditions were determined according to manufacturer’s instructions. Data on gene expression were normalized according to data from the *HPRT* housekeeping gene. Genes with uninterpretable amplifying curves were excluded from the analysis.

### Ethics statement

Mice were bred and maintained according to the French Ministry of Agriculture and European institutional guidelines (appendix A STE n°123). Animals were killed by cervical dislocation. Experiments were performed according to national regulations of the French Ministry of Agriculture and was specifically approved (ID approval N° 34118) by the regional ethics committee of Languedoc-Roussillon (Comité Régional d’Ethique sur l’Expérimentation Animale- Languedoc-Roussillon), France.

### Statistical analyses

For all quantitative analyses, a minimum of three independent experiments were performed. Student’s *t*-test were performed to analyze unpaired data.

## Supporting information

S1 Table List of genes analyzed in the PCR array. (XLSX)

S1 Fig Trypan blue assay (supernatant + adherent cells) showed that cell viability is not affected in USUV-infected astrocytes at 6 dpi at MOI of 0.1 and 2. Staurosporin-treated cells (1 μm for 6 hours) are used as cell death control. (***p<0.001).
(TIF)
