# Supplementary figures and images for: Deleterious effect of Usutu virus on human neural cells
Source: PLoS Negl Trop Dis. 2017 Sep 5;11(9):e0005913. doi: 10.1371/journal.pntd.0005913 (PMC5600396; doi:10.1371/journal.pntd.0005913)

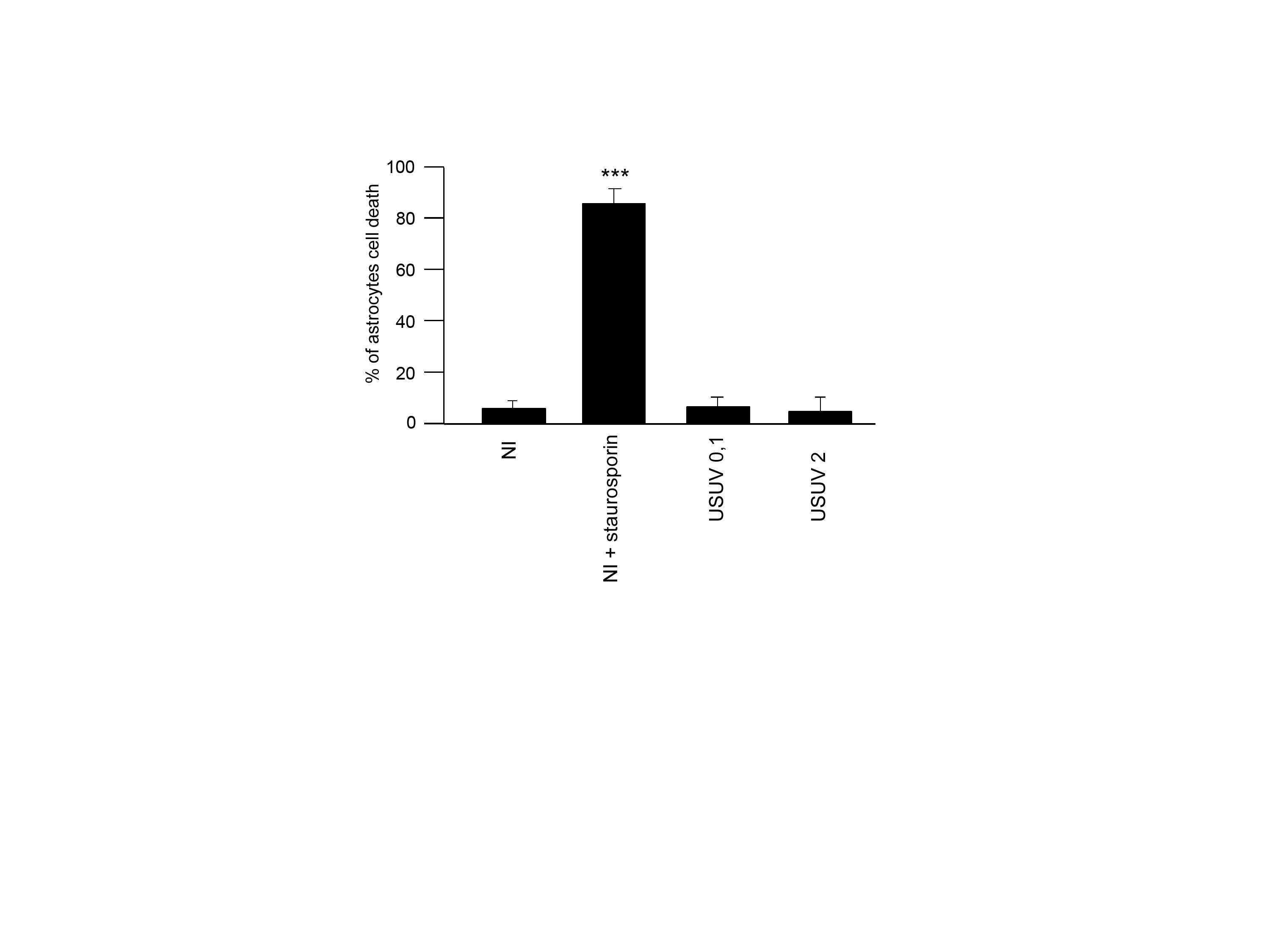

Supplement: S1 Fig — Staurosporin-treated cells (1 μm for 6 hours) are used as cell death control. (***p<0.001). (TIF) [file pntd.0005913.s002.tif]
